# Marek's disease virus infection of phagocytes: a *de novo in vitro* infection model

**DOI:** 10.1099/jgv.0.000763

**Published:** 2017-05-26

**Authors:** Pankaj Chakraborty, Lonneke Vervelde, Robert G. Dalziel, Peter S. Wasson, Venugopal Nair, Bernadette M. Dutia, Pete Kaiser

**Affiliations:** ^1^​ The Roslin Institute and R(D)SVS, University of Edinburgh, Easter Bush, Midlothian EH25 9RG, UK; ^2^​ Avian Oncogenic Virus Group, The Pirbright Institute, Guildford GU24 0NF, UK; ^†^​ Present address: Chittagong Veterinary and Animal Sciences University, Khulshi, Chittagong 4225, Bangladesh.; ^‡^​ Present address: MRC Technology, Crewe Road South, Edinburgh EH4 2SP, UK.

**Keywords:** MDV, *in vitro*, infection, macrophage, dendritic cell

## Abstract

Marek’s disease virus (MDV) is an alphaherpesvirus that induces T-cell lymphomas in chickens. Natural infections *in vivo* are caused by the inhalation of infected poultry house dust and it is presumed that MDV infection is initiated in the macrophages from where the infection is passed to B cells and activated T cells. Virus can be detected in B and T cells and macrophages *in vivo,* and both B and T cells can be infected *in vitro*. However, attempts to infect macrophages *in vitro* have not been successful. The aim of this study was to develop a model for infecting phagocytes [macrophages and dendritic cells (DCs)] with MDV *in vitro* and to characterize the infected cells. Chicken bone marrow cells were cultured with chicken CSF-1 or chicken IL-4 and chicken CSF-2 for 4 days to produce macrophages and DCs, respectively, and then co-cultured with FACS-sorted chicken embryo fibroblasts (CEFs) infected with recombinant MDV expressing EGFP. Infected phagocytes were identified and sorted by FACS using EGFP expression and phagocyte-specific mAbs. Detection of MDV-specific transcripts of ICP4 (immediate early), pp38 (early), gB (late) and Meq by RT-PCR provided evidence for MDV replication in the infected phagocytes. Time-lapse confocal microscopy was also used to demonstrate MDV spread in these cells. Subsequent co-culture of infected macrophages with CEFs suggests that productive virus infection may occur in these cell types. This is the first report of *in vitro* infection of phagocytic cells by MDV.

## Abbreviations

BAC, bacterial artificial chromosome; BMDC, bone marrow-derived dendritic cell; BMM, bone marrow-derived macrophage; CEF, chicken embryo fibroblast; DC, dendritic cell; MD, Marek’s disease; MDV, Marek’s disease virus.

## Introduction

Marek’s disease (MD) is a highly infectious and economically important oncogenic disease of chickens caused by the lymphotropic alphaherpesvirus Marek’s disease virus (MDV) (*Gallid alphaherpesvirus 2*). *G herpesvirus 2* belongs to the genus *Mardivirus* within the *Alphaherpesvirinae* subfamily. The ‘Cornell model’ of MDV infection [1] proposes that, *in vivo*, infection takes place when airborne cell-free virus wrapped in dander enters the respiratory tract and is engulfed by phagocytic cells, which carry the virus to the spleen and other lymphoid tissues. Virus is then thought to pass to lymphocytes where it causes lytic infection of B lymphocytes and lytic or latent infection in T cells. Infected T cells are thought to play a crucial role in the spread of virus to the various visceral organs and peripheral nerves, where proliferating T cells cause pathological lesions. The predominant role of lymphocytes in this disease has led to extensive studies of these cells. MDV-infected B and T lymphocytes can be readily detected *in vivo* [2], and *in vitro* models of lymphocyte infection were described by Calneck *et al.* [3] and more recently by Schermuly *et al.* [4]. However, the role of innate immune cells, in particular phagocytes, in MDV infection remains unclear.

Phagocytes are known to play crucial roles in limiting pathogen replication at initial stages and in the induction of adaptive responses in later stages [5]. There is good evidence that macrophages have a role in MDV pathogenesis. Peritoneal macrophages isolated from MDV-infected chickens have been shown to inhibit the formation of MDV plaques *in vitro* [6]. Furthermore, peritoneal macrophages from MD-susceptible chickens showed more phagocytic activity and plaque-inhibiting activity than those of resistant chickens following MDV infection *in vivo* [7]. Macrophages release NO (nitric oxide) through the increased activity of iNOS (inducible NO synthase), and NO showed inhibitory effects on *in vitro* and *in vivo* replication of MDV in the cytolytic and latent phase of MDV infection [8, 9]. Despite these studies, direct evidence of *in vivo* infection of macrophages by MDV was not demonstrated until the early 2000s [10]. However, several attempts to directly infect blood- or bone marrow-derived macrophages *in vitro* were not successful [10–12]. This led to the hypothesis that macrophages require *in vivo* conditions for infection by MDV [10]. Although macrophage infection can be studied *in vivo*, it is not always convenient for many laboratories around the world to carry out *in vivo* MDV-infection studies due to the lack of facilities, as well as the risk of spreading MDV. Developing an *in vitro* model to study MDV–macrophage interactions is therefore an important goal. Furthermore, differentiation and subsequent characterization of chicken bone marrow-derived dendritic cells (BMDC) *in vitro* [13] provides the opportunity to explore their interaction with MDV. The aim of this study was to establish an *in vitro* model of MDV infection of macrophages and DCs to enable detailed investigations of virus interactions in these cells.

## Results

### Chicken embryo fibroblast (CEF) cultures naturally contain macrophage-lineage cells

**Fig. 1.**
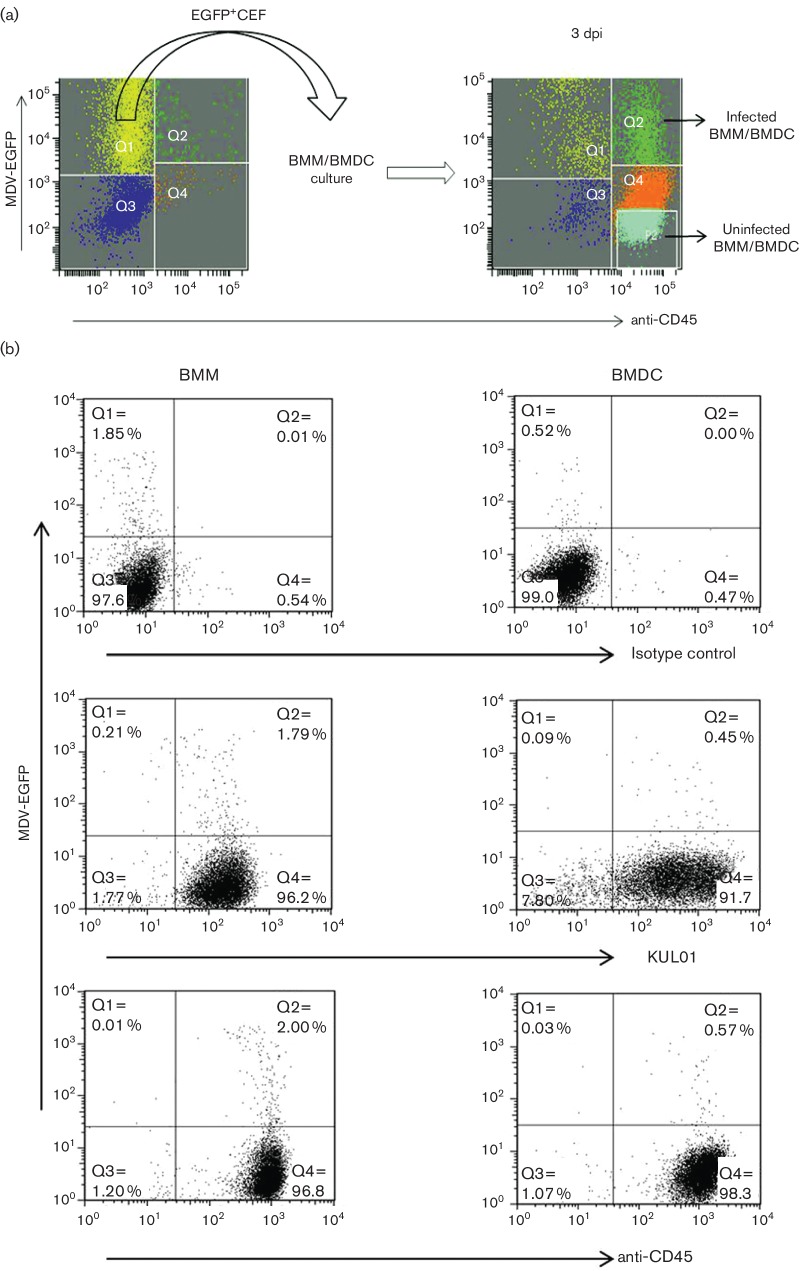
The *in vitro* infection of phagocytes with MDV. (a) The overall infection model. On the day of infection of phagocytes, MDV-infected CEFs were stained for CD45 expression to detect infected macrophages (Q2 in left plot) and EGFP^+^CD45^-^CEFs were sorted (left panel) and added to the bone marrow-derived macrophage (BMM) or bone marrow-derived DC (BMDC) culture. After 3 days in culture, infected and uninfected BMMs or BMDCs (EGFP^+^CD45^+^) were sorted (right panel). (b) Flow cytometric characterization of *in vitro-*infected BMMs and BMDCs. Chicken bone marrow-derived phagocytic cells were cultured with CSF-1 (for BMM) and with CSF-2 and IL-4 (for BMDC) for 4 days and then co-cultured with pre-sorted EGFP^+^CEF at a ratio of 1 : 5 (CEF:BMM/BMDC). Three days post-infection (p.i.), live cells were analysed for the surface expression of KUL01 and CD45 in BMM and BMDC. Gr 13.1 (class IgG1) was used as an isotype control antibody. Anti-CD45 antibody was used to detect the phagocytes via an AF647-tagged secondary antibody. Infected phagocytes were detected by double fluorescence of CD45 and EGFP (encoded with MDV). Data are shown as representative of two independent experiments for both BMM and BMDC. Distribution of cells: Q1, infected CEF; Q2, infected macrophage/DC; Q3, uninfected CEF; Q4, uninfected macrophage/DC; P2, sorting zone for uninfected macrophage/DC.

In order to study the infection of macrophages and DCs with MDV, we used a recombinant MDV which expresses EGFP under control of the murine phosphoglycerol kinase promoter, independently of viral gene expression. Prior to infection studies, EGFP^+^ MDV-infected CEFs were stained with the macrophage-specific antibody KUL01 in order to determine whether the infectious virus preparation contained macrophage-lineage cells. Flow cytometric analysis revealed that MDV-infected CEFs contained a significant percentage (8 %) of KUL01^+^ cells and approximately 0.1 % of these cells were infected with MDV (see Fig. S1, available in the online Supplementary Material). As macrophages are known to express the CD45 marker [14], we used an anti-CD45 antibody to remove macrophages and any other cells of leukocyte origin from the infected CEF cultures prior to their use to infect macrophages and DC cultures. Notably, staining with anti-CD45 was of a higher and more uniform intensity than that of KUL01 (Fig. 1b), providing an advantage during cell sorting. Selection of CD45^-^EGFP^+^CEFs by cell sorting prior to infection of macrophages and DCs ensured that the infectious inoculum did not contain macrophages or cells of other lymphoid origin.

### Phagocytes were infected *in vitro* by MDV

Both bone marrow-derived macrophages (BMMs) and bone marrow-derived DCs (BMDCs) were co-cultured with MDV-infected CD45^-^EGFP^+^CEFs at the same ratios (1 : 5) as shown in Fig. 1(a). Three days p.i., flow cytometric analysis of live cells demonstrated that BMMs and BMDCs could be infected with MDV *in vitro*. Similar percentages of EGFP^+^cells were observed when cultures were stained with KUL01 and CD45 (Fig. 1b). The proportion of infected BMMs, as shown by KUL01 and CD45 staining, was around 2 %, whereas the percentage was much less in infected BMDCs where only around 0.5 % were infected (Fig. 1b).

### Visualization of MDV replication in phagocytes

**Fig. 2.**
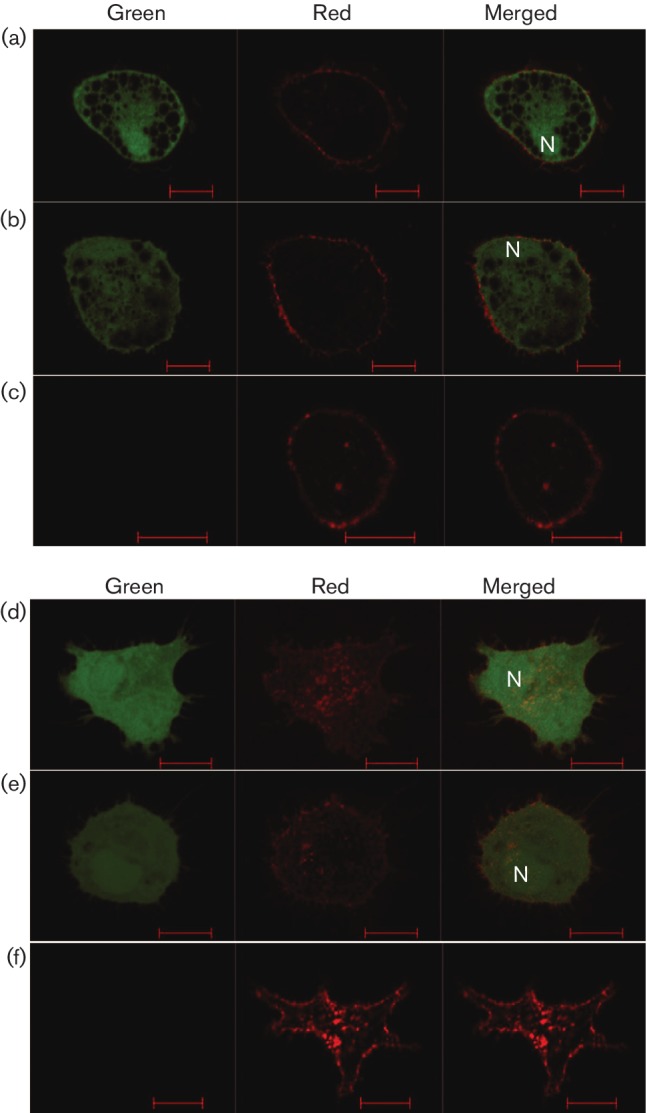
Visualization of infected and uninfected BMMs and BMDCs. Phagocytic cells were infected *in vitro* with EGFP^+^CEFs. Three days p.i., BMMs and BMDCs were sorted following staining with anti-CD45 and examined under confocal microscopy for (a, b) infected BMMs and (c) uninfected BMMs, as well as for (d, e) infected DCs and (f) uninfected DCs. Green channel: cells examined for the expression of EGFP-encoded MDV; red channel: cells examined for the expression of CD45 (AF647); merged channel: cells examined for combined expression of green and red. N, nucleus. Scale bar, 10 µm.

Following infection, BMMs and BMDCs were examined by confocal microscopy to detect whether EGFP expression was intracellular or surface-bound. Fig. 2(a, b) shows that in the infected macrophage, the EGFP signal is detected throughout the cytoplasm and in the nucleus but not in vacuoli. Fig. 2(d, e) shows that in the DCs, EGFP was found to be dispersed throughout the cytoplasm and in the nucleus. The localization of EGFP was further evaluated using Z-stack analysis to explore the exact site of EGFP expression, confirming that EGFP was present in the nucleus and not simply overlying it (data not shown). If the macrophages had simply phagocytosed infected CEFs and were not infected, EGFP would not have been present in the nucleus. The presence of EGFP in both the nucleus and the cytoplasm indicates transcription of virus genome and, hence, MDV infection of these cells (Fig. 2a, b). Uninfected BMMs showed only the expression of CD45 (Fig. 2c). No EGFP was detected in uninfected DCs (Fig. 2f).

### Detection of viral gene expression in phagocytes

**Fig. 3.**
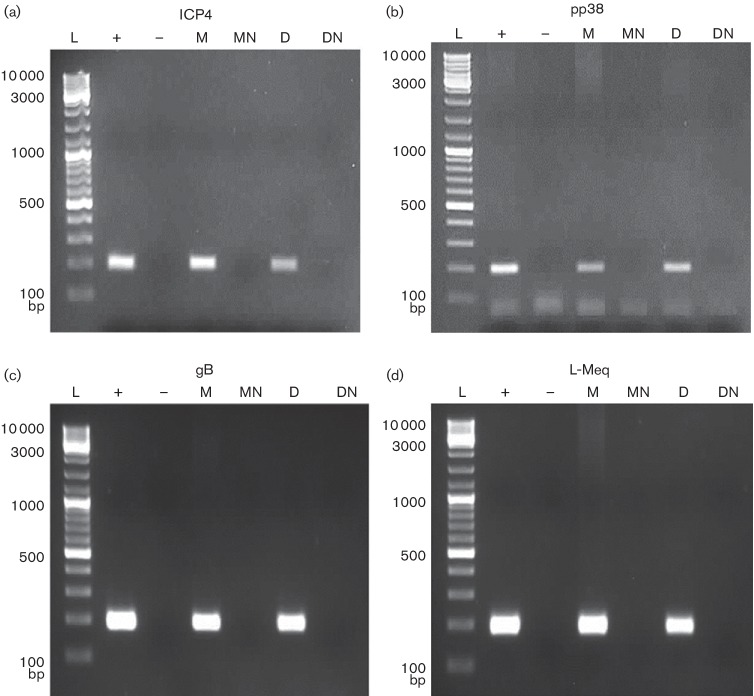
Detection of MDV transcripts in BMMs and BMDCs infected with MDV *in vitro*. BMMs and BMDCs were infected *in vitro* with EGFP-expressing MDV. After 3 days, EGFP-positive cells were sorted and RT-PCR was carried out for the detection of (a) immediate early ICP4 (200 bp), (b) early pp38 (198 bp), (c) late gB (193 bp) and (d) MDV-specific l-Meq (200 bp) transcripts. L, ladder; +, positive control MDV-infected CEFs; −, negative control, nuclease-free H_2_O; M, infected BMMs (cDNA); MN, infected BMMs no-RT control (DNase-treated RNA); D, infected BMDCs (cDNA); DN, infected BMDCs no-RT control (DNase-treated RNA).

RT-PCR was performed to detect the transcription of virus genes in MDV-infected CEFs, BMMs and BMDCs. Herpesvirus-specific immediate early (ICP4), early (pp38) and late (gB) genes, as well as the MDV-specific oncogene (Meq), were expressed (Fig. 3) in MDV-infected BMMs and BMDCs. Transcription of all virus genes in MDV-infected BMMs and DCs was compared to those in virus-infected CEFs, which were used as a positive control. DNase-treated RNA from both infected BMMs and DCs was used in PCRs as no-RT controls. The absence of bands in these samples (MN and DN) confirmed the transcription of the virus genes in MDV-infected BMMs and BMDCs (Fig. 3).

### Modes of MDV transmission between cells

MDV is strictly cell-associated *in vitro*, and transmission of MDV between cells should therefore occur through cell-to-cell contact. However, in the case of macrophages, the possibility of direct infection following phagocytosis of MDV-infected CEFs cannot be ruled out. To determine the possible mode(s) of MDV infection of macrophages, time-lapse microscopic imaging was carried out from the day of infection of BMMs with MDV-infected CEFs. Analysis of Video S1 (in the Supplementary Materials) reveals that EGFP^+^ particles are moving around within the cellular vacuoles, suggesting internalization of MDV by macrophages, which could result in their infection. However, Video S2 shows a large, infected macrophage-like cell demonstrating intercellular connections with two other small-sized cells and also green cellular processes emerging from cell surfaces, suggesting a cell-to-cell mode of transmission of the virus in these cell types. These EGFP^+^ cellular projections could be an indication of actin-mediated transmission of MDV, as previously described [15].

### MDV–macrophage interaction results in a productive infection

**Fig. 4.**
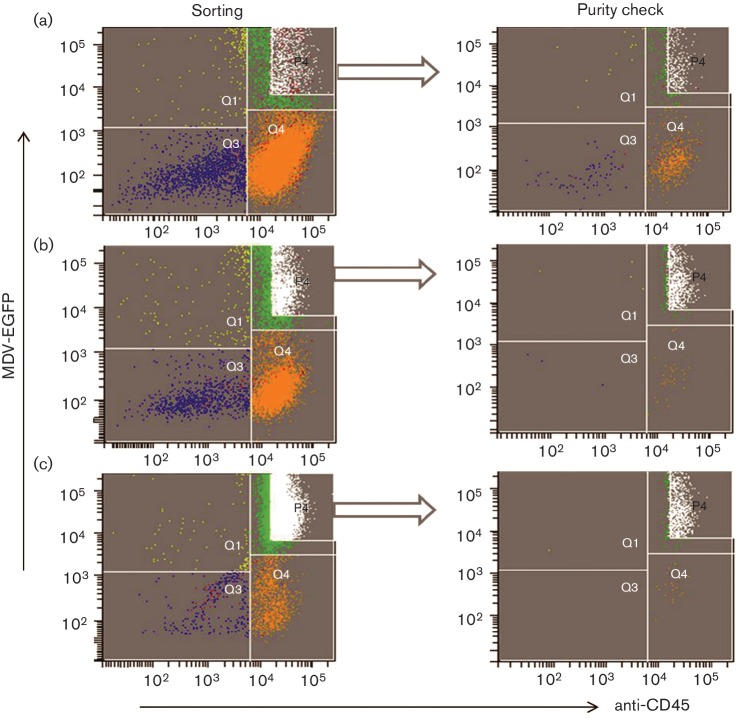
Triple-sorting of MDV-infected BMMs with corresponding purity. (a) In the first sort, 1.28×10^6^ cells were sorted based on the gate P4 and the analysis revealed the presence of contaminant cells in Q1, Q3 and Q4. (b) Sorted cells (from P4) were re-sorted and contamination with infected CEFs (Q1); four events per 1000 infected macrophages were identified. (c) After sorting BMMs for the third time, only one infected CEF (Q1) per 1000 infected macrophages was detected. These triple-sorted infected BMMs were added to CEF cultures. The *y*-axis shows the fluorescence of intracellular EGFP-encoded MDV and the *x*-axis shows the fluorescence of AF 647-tagged anti-CD45. Distribution of cells in sorting plots: Q3, uninfected CEF; Q1, infected CEF; Q4, uninfected macrophage; Q2, infected macrophage; P4, sorting zone for infected macrophage.

**Fig. 5.**
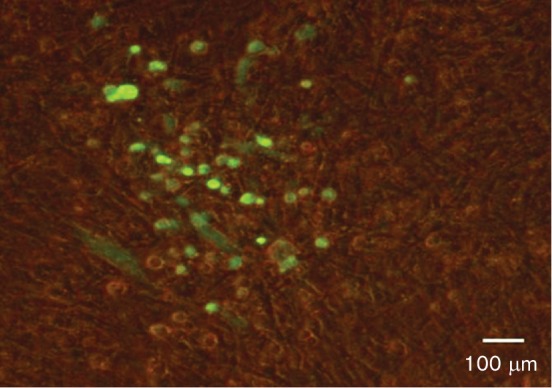
Formation of plaques following infection of CEFs with MDV-infected BMMs. To investigate the productive or abortive nature of MDV-BMM infections, CEFs were re-infected with MDV-infected BMMs. In order to reduce the number of contaminant-infected CEFs, MDV-infected BMMs were sorted three times and freshly cultured CEFs were infected with these triple-sorted infected BMMs. Five days p.i., plaques were visualized based on EGFP (encoded with MDV) using a fluorescence microscope.

To determine whether the *in vitro* MDV–macrophage infection is productive or abortive, CEFs were ‘re-infected’ with MDV-infected BMMs. EGFP^+^BMMs were co-cultured with uninfected CEFs and plaques were observed at 5 days p.i. in CEF monolayers. However, FACS analysis showed that the EGFP^+^BMMs contained a small percentage of contaminant EGFP^+^CEFs (<2 %, Fig. S2). EGFP^+^BMMs were therefore triple sorted. As shown in Fig. 4(a–c), this reduced the contaminating CEFs to 0.1 %. These triple-sorted infected BMMs were added to fresh uninfected CEF cultures and incubated for 5 days. Addition of infected BMMs to CEFs resulted in the formation of around 50 plaques, as illustrated in Fig. 5. Based on the percentage of contaminating CEFs, only 10 of these might be attributable to CEFs thus indicating the likely production of infectious virus in the BMMs.

## Discussion

As MDV is a lymphotropic virus, the majority of the studies on virus–cell interactions in MDV have been conducted on lymphocytes. However, innate immune cells such as macrophages are also associated with the early stages of the MDV life cycle [1]. Although macrophages can be infected with MDV *in vivo* [10, 16], extensive MDV–macrophage interaction studies are not possible with the small numbers of infected macrophages obtained in *in vivo* experiments. This research therefore aimed to establish a new *in vitro* MDV infection model of BMMs and BMDCs. MDV remains strictly cell-associated in all cultured cells and, unlike other herpesviruses, its infectivity cannot be recovered from supernatants or even from cell lysates (reviewed in [17]). Although fully infectious cell-free viruses can be processed and purified from feather follicle epithelium and used for *in vivo* infection studies, the virus titre is not sufficiently high for use in *in vitro* studies (unpublished observations). MDV-infected CEF cultures are widely used as input material for both *in vitro* and *in vivo* infection. However, we noted that (Fig. S1), in addition to cells of the fibroblast lineage, these cultures also contained cells of the macrophage lineage that are readily identified by KUL01 staining. The observation that the CEF cultures contained macrophages is not surprising as these are prepared from 9- to 11-day-old whole chicken embryos, and embryonic macrophages have been shown to appear as early as 2.5–4.5 days in developing embryos [18]. Furthermore, the production of iNOS, an indicator of the presence of macrophage-like cells, has been reported in CEF cultures [8]. Therefore, EGFP^+^MDV-infected CD45^-^CEFs were isolated by FACS before attempting to infect BMM and BMDC *in vitro*.

Following co-culture, infected and uninfected BMMs were characterized by flow cytometry at 3 days p.i. and the successful *in vitro* infection of BMM with MDV was observed. Similarly, we were also able to show that BMDC could be infected *in vitro.* As MDV infection of DCs, either *in vivo* or *in vitro*, has not been reported previously, our study is the first demonstration of infection of DCs by MDV. Despite the same infection ratio, the percentage of MDV-infected BMMs was higher than that of DCs. A possible explanation is that the BMMs were cultured in chicken CSF-1 whereas the DCs were cultured in chicken IL-4 and chicken CSF-2. This will to lead to different transcription profiles in the two cell types, which may affect their susceptibility to infection. It is not known whether DCs and macrophages have the same susceptibility to infection *in vivo*, and further work is needed to understand the difference in susceptibility.

Confocal microscopy of live cells cultured with EGFP-expressing infected CEFs showed the presence of EGFP in both the cytoplasm and nuclei of BMMs and BMDCs. This provides strong support that these cells were infected with MDV, rather than from the effect of phagocytosis of MDV-infected CEFs, as viruses in phagosomes are known to be rapidly degraded (reviewed in [19]). While cytoplasm-restricted EGFP expression could perhaps be argued as an outcome of phagocytic internalization of virus-infected cell(s), expression of virus-encoded EGFP in the nucleus clearly gives a strong indication of virus replication in the BMM.

MDV-specific immediate early (ICP4), early (pp38) and late (gB) genes and localization of ICP4 to the nucleus of *in vivo*-infected KUL01^+^ cells was used to confirm MDV infection of macrophages [10]. In our study, we demonstrated MDV-encoded transcripts of ICP4, pp38, gB and Meq in EGFP^+^ BMMs and BMDCs, providing further evidence of MDV infection of these cells.

Time-lapse microscopy imaging was used to investigate possible modes of MDV transmission to BMMs on the day of infection. Video S1 shows green particles in intracellular vacuoles of the BMM. In addition to phagocytosing small particles and microbes, macrophages also capture apoptotic bodies produced by dying cells by the process of efferocytosis. Whilst the processes of phagocytosis and efferocytosis can both lead to the destruction of engulfed material following fusion with lysosomes, efferocytosis has been recognized as a way in which pathogens aid their dispersal [20]. Furthermore, there is evidence that herpesviruses use phagocytic processes to enter the cells for initiating infection, possibly by fusion of the viral and vesicle membranes [21, 22]. Our data provide support for the hypothesis that BMMs are infected via one of these pathways, but further work is needed to show that this is the case.

Video S2 illustrates the potential cell-to-cell transmission of MDV between BMMs. As a strictly cell-associated virus, cell-to-cell modes of transmission are to be expected. However, the exact mechanism or molecular events of cell-to-cell transmission of MDV is not fully understood (reviewed in [17]). Video S2 shows potential cellular connections with green cellular projections from and between cells. These projections are most likely actin microfilaments [15]. During entry of pseudorabies virus, the viral protein US3 plays a crucial role in the generation of actin-containing cellular extensions, which is followed by trafficking of virions in phagosome-like vesicles [21, 23, 24]. Schumacher *et al.* [25] reported that polymerization of the actin cytoskeleton is required for the effective cell-to-cell spread of MDV in chicken embryo cells *in vitro*, and the US3 orthologue of MDV plays a similar role. Clement *et al.* [21] showed that HSV-1 can induce actin- or tubulin-containing structures by which virions project towards adjacent cells – a route of transmission that may also be applicable to MDV.

In order to determine the productive or abortive nature of MDV-BMM infection, attempts were made to re-infect CEFs with EGFP^+^BMMs *in vitro*. Barrow *et al.* [10] reported that the *in vivo* MDV-macrophage infection is an abortive infection as infected macrophages failed to produce plaques in CEF cultures. In the present study, plaques were observed in CEF cultures following co-culture with triple-sorted infected BMMs, suggesting the productive nature of MDV-BMM infection. However, further work is needed to confirm this as, even after triple sorting, a very small number of infected CEFs could be detected in the infected BMMs and further sorting resulted in compromised viability of the macrophages.

Taken together, we present a novel *in vitro* model for the infection of phagocytes with MDV. This model will enable further studies into MDV–phagocyte interactions and in determining the cellular basis of resistance to MD. In addition, the model may be used for other avian viruses that may be spread in the chicken via macrophages such as infectious bronchitis virus and avian influenza virus.

## Methods

### Chickens and the virus

Layer chicken line J, an intercross bred from nine lines, originally inbred from Brown Leghorn chickens at the Poultry Research Centre, Edinburgh, was bred and conventionally raised at The Roslin Institute (www.narf.ac.uk/chickens/lines). Animals were housed in premises licensed under a UK Home Office Establishment License within the terms of the UK Home Office Animals (Scientific Procedures) Act 1986. Housing and husbandry complied with the Code of Practice for Housing and Care of Animals Bred, Supplied or Used for Scientific Purposes and were overseen by the Roslin Institute Animal Welfare and Ethical Review Board. Animals were culled by schedule one methods authorized by the Animals (Scientific Procedures) Act 1986.

The virus, CVI988 UL41 EGFP, was generated from a bacterial artificial chromosome (BAC) construct of vaccine strain CVI988 (Rispens) of MDV serotype 1, in which the UL41 gene was replaced with EGFP under control of the murine phosphoglycerol kinase promoter [26]. UL41 is a non-essential gene for MDV replication, and a UL41-deletant mutant replicates as well as the parental strain *in vitro* [27]. The presence of EGFP will therefore indicate MDV replication.

### Cell cultures

CEFs were cultured from 9- to 11-day-old chicken embryos and cultured in T_175_ flasks at 38.5 °C with 5 % CO_2_ in CEF medium consisting of M-199 medium (Gibco) containing 10 % (v/v) tryptose phosphate broth (Invitrogen), 2.7 % (v/v) NaHCO_3_ (Sigma-Aldrich), 1 % (v/v) pen-strep (Sigma-Aldrich), 0.5 % (v/v) gentamycin (Sigma-Aldrich), 0.001 % (v/v) fungizone (amphotericin B, 250 µg ml^−1^) (Thermo Scientific), and 0.5–10 % (v/v) FBS (Gibco) depending on CEF confluency in culture flasks. The MDV-BAC virus was initially grown and propagated in CEF cultures as described previously [28]. MDV-infected CEFs were then grown in large numbers and pooled together to obtain a high virus titre. Pooled infected CEFs were resuspended in freezing media (FBS, RPMI-1640 and DMSO), aliquoted (250–500 µl per cryovial) and stored at −80 °C until further use.

Chicken bone marrow cells were isolated from 3- to 6-week-old birds and BMMs and BMDCs were cultured as described previously [13, 29]. Cells were cultured for 4 days in T_75_ flasks at 41 °C with 5 % CO_2_ using RPMI-1640 medium (Sigma-Aldrich) supplemented with 10 % heat-inactivated FBS (PAA) (for BMMs), 10 % heat-inactivated chicken serum (for BMDCs), 1 % l-glutamine and 0.1 % pen-strep. Recombinant chicken interleukin-4 (chIL-4) and granulocyte-macrophage colony-stimulating factor (chCSF-2 or GM-CSF) were added to the BMDC cultures at the optimal dilution of each cytokine, whereas recombinant chCSF-1 was added to the BMM cultures. In order to obtain approximately 1×10^7^ BMMs or BMDCs at harvest, bone marrow cells were seeded at a concentration of approximately 1×10^6^ cells ml^−1^.

### Co-culture infection experiments, FACS and flow cytometry

Due to the cell-associated nature of MDV, infected CEFs were used to infect phagocytes. Prior to the infection of phagocytes, previously frozen virus was propagated in large numbers in CEF cultures. On the day of phagocyte infection, infected CEFs were harvested by 2.5 % trypsin (diluted in PBS), pelleted by centrifugation (500 ***g*** for 5 min) and resuspended in FACS buffer (PBS and 1 % BSA). Immunofluorescent staining of infected CEFs was carried out as described previously [30] using anti-CD45 (clone AV53, isotype IgG1; The Pirbright Institute) and a goat anti-mouse IgG1 conjugated with AF 647 as a secondary antibody. Gr 13.1 (ovine NKp46; kindly provided by Dr Timothy Connelley, The Roslin Institute) was used as isotype control. EGFP^+^CD45^-^CEFs were sorted using the FACSAriaTM III cell sorter (BD Biosciences). Data analyses were carried out using FACSDiva v 6.1.3 software.

BMMs and BMDCs were infected with 2×10^6^ sorted infected CEFs on day 4 of culture in T_75_ flasks at an infection ratio of 1 : 5 (CEF:BMM or BMDC) in RPMI-1640 medium containing 2–10 % FBS (Gibco; serum percentage was determined according to the confluency of CEF in culture flask), 1 % pen-strep and 1 % l-glutamine. In addition, the medium for BMDCs was supplemented with 5 % chicken serum. Co-cultured cells were incubated at 41 °C with 5 % CO_2_ for 3 days and harvested for downstream experiments, such as flow cytometry or cell-sorting. For flow cytometry, cells were harvested with 100 mM EDTA in PBS, pelleted by centrifugation and resuspended in PBS containing 1 % BSA and 0.1 % sodium azide. Immunofluorescent staining was carried out using a macrophage marker (clone KUL01, isotype IgG1; Southern Biotech) and anti-CD45. KUL01 was recently identified as a mannose receptor [31]. Cells were stained for flow cytometric analysis as described above and analysed using a FACSCalibur (BD Biosciences). Viable cells were gated based on 7-AAD (7-aminoactinomycin D, Life Technologies) staining, and the resulting data were analysed with FlowJo software.

### RT-PCR

RNA samples were extracted using RNeasy Mini Kits (Qiagen) and treated with DNase (Ambion Turbo DNA-free Kits, Life Technologies). Reverse transcription of RNA was carried out using Superscript III reverse transcriptase (Invitrogen) as per the manufacturer’s instructions. PCRs were performed on a Mastercycler Thermo cycler (Eppendorf) using recombinant *Taq* DNA polymerase (Invitrogen). Primers used for ICP4, pp38, gB and l-Meq are listed in Table 1. The reaction mixtures contained 10× PCR buffer minus Mg^2+^, 50 mM MgCl_2_, 10 mM dNTP mixture, 10 µM forward primer, 10 µM reverse primer, 0.6–0.8 µl *Taq* polymerase (5 units µl^–1^), 20–25 ng cDNA template and H_2_O. Cycling conditions for PCRs were: denaturation at 95 °C for 3 min, amplification with 30 cycles of 94 °C for 1 min 59 °C for 1 min, and 72 °C for 30 s. The PCRs were extended for 6 min at 72 °C.

**Table 1.** Primers used for PCR

Primers	Sequence (5′−3′)	Ensemble accession no.
ICP4 F	GGTGATCCTGGCCTTGTAAA	M75729
ICP4 R	TGGGTGGATTTAATGGGAGA	
pp38 F	GCTAACCGGAGAGGGAGAGT	M73484
pp38 R	TCCGCATATGTTCCTCCTTC	
gB F	CCGCTCTGTGTTTCCGTATT	AY129968
gB R	CTTGACTGGAAGGCTTGCTT	
l-Meq F	GTCGACTTCGAGACGGAAAA	AB033119
l-Meq R	GCAGCTCTTCACATGCTTCA	

ICP4, infected cell protein 4; pp38, phosphoprotein 38; gB, glycoprotein B; l-Meq, long isoform of Meq (MDV EcoRI-Q).

### Confocal microscopy and time-lapse imaging

Following co-culture of infected CEFs and BMMs or BMDCs, the infected and uninfected BM cells were sorted, pelleted (1200 ***g*** for 5 min) and resuspended in 1 ml co-culture media. The cells were placed in sterile chamber slides mounted on borosilicate cover glass (Nunc, ThermoFisher Scientific) and incubated at 41 °C for at least 2 h. Once settled, cells were examined under a confocal microscope (LM710 Confocal AxioObserver, Zeiss) with objective ×63/1.40 Oil DIC. Captured images were analysed with Zen2011 image processing software.

For time-lapse imaging, BMMs were cultured in sterile chamber slides, at a concentration of 1×10^6^ cells per chamber, and infected on day 4 with 1×10^5^ sorted infected CEFs per chamber. Following infection, cells were imaged using a Zeiss LSM710 confocal microscope, maintaining optimal culture conditions with images captured every 10 s. Images from a 10 min experiment were combined to create a movie using Zen2011 image processing software.

### Re-infection of CEFs

BMMs were cultured for 4 days and then infected with sorted EGFP^+^CEFs as mentioned above. After 3 days of co-culture, infected BMMs were sorted by selection of CD45^+^EGFP^+^ cells and added to fresh CEF cultures with co-culture medium containing 0.5 to 1 % FBS (depending on CEF confluency) and CSF-1 (4 %). Cells were incubated at 41 °C with 5 % CO_2_. Transmission of virus from infected BMMs to CEFs was determined by quantification of fluorescent plaques formed in the CEF monolayers.

## Supplementary Data

1665 Supplementary File 2

## Supplementary Data

1792 Supplementary File 3

## Supplementary Data

504 Supplementary File 4
