# Supplementary material for: Marek's disease virus infection of phagocytes: a de novo in vitro infection model
Source: J Gen Virol. 2017 May 26;98(5):1080–8. doi: 10.1099/jgv.0.000763 (PMC5656796; doi:10.1099/jgv.0.000763)
Supplement: Supplementary File 4 [file jgv-98-1080-s003.pdf]

Supplementary figures

Figure S1. CEFs infected with EGFP$^+$MDV contain cells of the macrophage lineage which are infected with MDV.

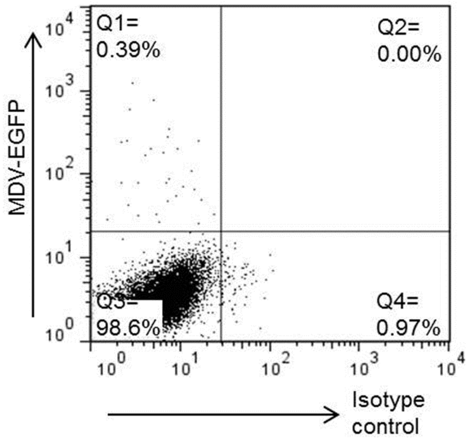

Figure S2. Analysis of MDV-infected BMM (EGFP$^+$CD45$^+$) reveals that a single round of sorting does not remove all EGFP$^+$CD45$^-$ CEFs present in the inoculum.

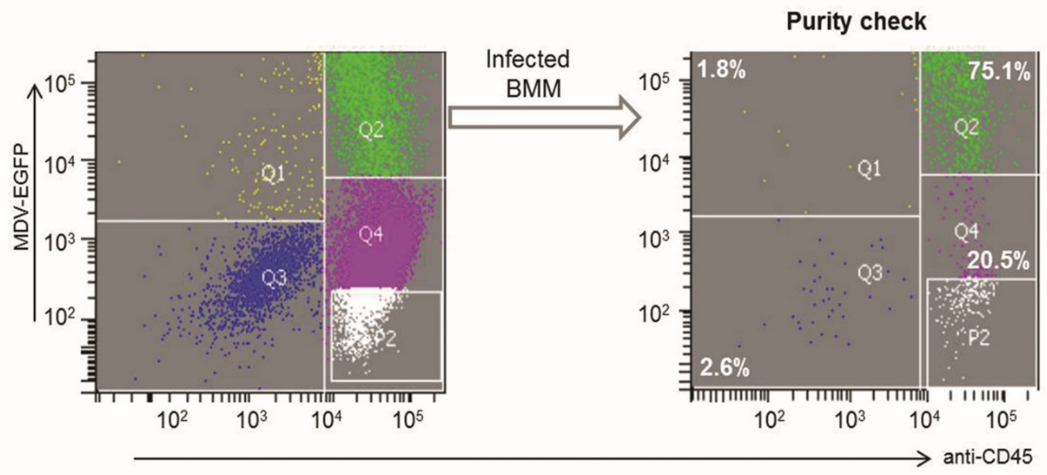

Supplementary figure legends

Fig. S1. Presence of macrophages in CEF cultures and staining pattern of macrophages. (a) CEF cultures naturally contain macrophage-lineage cells. In order to detect macrophage-lineage cells in CEF cultures, previously frozen EGFP expressing MDV-infected CEF were thawed and stained with a mouse monoclonal antibody to chicken macrophages, KUL01 followed by secondary antibody conjugated with AF647. Flow cytometric analysis shows that the CEF culture contains 8% macrophages including a small percentage (0.07%) of $EGFP^{+}MDV^{-}$infected macrophages.

Fig. S2. Purity of infected BMM. *In vitro* derived BMM were infected for 3 days with MDV infected CEF expressing EGFP. Virus positive BMM were selected by cell sorting for $CD45^{+}EGFP^{+}$ cells (left). Sorted cells were reanalysed for the presence of contaminant cells by placing 1000 cells in the same sorting plot (right). Whilst the majority of cells were $CD45^{+}EGFP^{+}$ (Q2, the sorted population consistently contained around 1-2% infected CEF (Q1) as well as $EGFP^{-}$CEF (Q3) and uninfected BMM (Q4). P2 indicates the sorting zone for uninfected macrophage.
